# Health and Capital

**Published:** 1876-06

**Authors:** 


### HEALTH AND CAPITAL.

Political economists understand that Health is Capital as much as money or lands, and its preservation is vastly more important. Lost money or estates may be recovered by industry, when the health is unimpaired. But when health goes all is lost. In view of these facts how insane most men seem. We speak particularly of Americans, for in other countries men are usually satisfied with a competency, and when that is obtained, spend the rest of their lives in rational enjoyments. Your gaunt, nervous, dyspeptic, anxious American is an overworked "pack horse," whose chief desire is to make money, without any definite idea of what he shall do with it. To this end he frequently sacrifices both "life and health. In the language of Aunt Betsy Trotwood he is "blind, blind." He can neither see the true aim of life, nor appreciate its glorious gifts. Such a man deserves our commiseration, though he counts his hoarded wealth by millions. His riches comprise all there is of him. They bring him neither true happiness nor sincere respect. When the shadow of death falls on the threshold, and he sees when too late the sacrifices made to Mammon, how gladly he would give up all he has to be restored to health, with opportunity to profit by his sad experience.

But life is a road we travel but once. Our reason teaches us what we should do at every stage. If we fail to heed these counsels by neglect of health or duty, we suffer when the inevitable day of reckoning comes. It would seem that no one but an idiot or a lunatic would venture to shatter and destroy the beautiful "Temple" that God has given him for his earthly abode. But still we see men every day who are regarded as mentally sound and rational, who, by bad habits, are deliberately and certainly undermining health, strength, and life itself, with all the deliberation and even dignity of a high mission honorably performed.

